# Nutrient and Bioactive Fraction Content of *Olea europaea* L. Leaves: Assessing the Impact of Drying Methods in a Comprehensive Study of Prominent Cultivars in Morocco

**DOI:** 10.3390/plants13141961

**Published:** 2024-07-17

**Authors:** Salah Chaji, Walid Zenasni, Rachida Ouaabou, El Amine Ajal, Rachid Lahlali, Marie-Laure Fauconnier, Hafida Hanine, Marko Černe, Igor Pasković, Othmane Merah, Aadil Bajoub

**Affiliations:** 1 Laboratory of Food and Food By-Products Chemistry and Processing Technology, National School of Agriculture in Meknès, km 10, Haj Kaddour Road, B.P. S/40, Meknès 50001, Moroccorlahlali@enameknes.ac.ma (R.L.); 2 Laboratory of Bioprocess and Bio-Interfaces, Faculty of Science and Techniques, University Sultan Moulay Slimane, B.P. 523, M’Ghila, Beni Mellal 23000, Morocco; 3 Laboratory of Research and Development in Engineering Sciences, Faculty of Sciences and Techniques Al Hoceima, Abdelmalek Essaadi University, B.P. 34, Al-Hoceima 32003, Morocco; 4 UPR of Pharmacognosy, Faculty of Medicine and Pharmacy of Rabat, Mohammed V University, B.P. 6203, Rabat 10000, Morocco; 5 Laboratory of Chemistry of Natural Molecules, Gembloux Agro Biotech, University of Liege, 5030 Gembloux, Belgium; marie-laure.fauconnier@uliege.be; 6 Institute of Agriculture and Tourism, Karla Huguesa 8, 52440 Poreč, Croatia; marko@iptpo.hr (M.Č.); paskovic@iptpo.hr (I.P.); 7 Laboratoire de Chimie Agro-Industrielle (LCA), Institut National de Recherche Agronomique et Environnement (INRAE), Institut National Polytechnique de Toulouse (INPT), Université de Toulouse, 31030 Toulouse, France; 8 Département Génie Biologique, Institut Universitaire de Technologie Paul Sabatier, Université Paul Sabatier, 32000 Auch, France; 9 Department of Analytical Chemistry, Faculty of Science, University of Granada, Ave. Fuentenueva s/n, 18071 Granada, Spain

**Keywords:** olive leaves, valorization, drying, nutrient compounds, bioactive compounds, liquid chromatography, Moroccan cultivars

## Abstract

This study explores the potential of olive leaves, long integral to Mediterranean traditional medicine, as a rich source of valuable compounds. The challenge lies in their considerable water content, hindering these compounds’ full valorization. Four drying methods (air-drying, oven-drying, freeze-drying and solar-drying) were investigated for their impact on nutrient and bioactive compound content in the leaves of four olive varieties (“Arbequina”, “Koroneiki”, “Menara” and “Picholine Marocaine”) cultivated in Morocco. In their fresh state, “Picholine Marocaine” exhibited the highest protein levels (6.11%), “Arbequina” had the highest phenolic content (20.18 mg gallic acid equivalents/g fresh weight (FW)), and “Koroneiki” and “Menara” were highest in flavonoids (3.28 mg quercetin equivalents/g FW). Specific drying methods proved optimal for different varieties. Oven-drying at 60 °C and 70 °C effectively preserved protein, while phenolic content varied with drying conditions. Air-drying and freeze-drying demonstrated effectiveness for flavonoids. In addition, an analytical approach using high-performance liquid chromatography and diode array detection (HPLC-DAD) was applied to investigate the effects of the different drying methods on the bioactive fraction of the analyzed samples. The results showed qualitative and quantitative differences depending on both the variety and the drying method used. A total of 11 phenolic compounds were tentatively identified, with oleuropein being the most abundant in all the samples analyzed. The freeze-dried samples showed the highest content of oleuropein in the varieties “Arbequina” and “Picholine Marocaine” compared to the other methods analyzed. In contrast, “Koroneiki” and “Menara” had higher oleuropein content when air dried. Overall, the obtained results highlight the importance of tailored drying techniques for the preservation of nutrients and bioactive compounds in olive leaves.

## 1. Introduction

Since ancient times, *Olea europaea* L. leaves and its derived extracts have been largely used all over the Mediterranean area in traditional medicine against various kinds of infectious and non-infectious diseases [1]. Recently, these traditional medicinal practices have captured significant attention worldwide, fueled by well-established scientific research findings supporting the health-promoting properties of olive leaves [2,3]. With the tremendous advancements in analytical techniques, coupled with rigorous in vitro and in vivo assays, substantial scientific evidence has been accumulated over the past decades regarding the therapeutic potential and functional proprieties of olive leaves and their extracts [4,5]. Overall, these natural products appear to be effective for the treatment and prevention of diabetes, skin problems and neurodegenerative and cardiovascular diseases [3,6]. These beneficial effects are mainly attributed to their phytochemical content, particularly phenolics and proteins, which possess various health-related proprieties [6,7,8].

Indeed, it has been found that the protein content in olive leaves varies between 5.04% and 12.40% depending on the variety [9,10]. Meanwhile, olive leaves’ phenolic fraction consists mainly of secoiridoids and their derivatives, and even other compounds such as flavonols and phenolic acids are also present in lower concentrations [6]. Of these antioxidant compounds, oleuropein, luteolin-7-*O*-glucoside and apeginin-7-*O*-glucoside are most abundant in olive leaves [6,11]. Given their richness in bioactive compounds and the fact that the pruning of olive groves in the Mediterranean produces 1 to 11 tonnes of olive leaves per hectare per year, they are currently considered a rich, renewable and promising source of valuable compounds with high potential for recovery and further application and valorisation in the food, cosmetic and pharmaceutical industries [12,13].

The utilization of olive leaves to recover functional components with interesting bioactivities, which can be further used by industries, has recently garnered tremendous attention due to the growing consumer awareness of sustainability and the global environmental regulations related to agri-food by-product valorization [14]. A typical recovery process involves the following five steps: (1) pre-treatment through drying and grinding the leaves; (2) diffusion extraction to solubilize the phenolic compounds in the extraction solvent; (3) concentration and purification of the liquid phase to isolate the targeted compounds and remove unwanted solutes; (4) evaporation of the solvent and its potentially recycling for further use; and finally, (5) drying of the extract to obtain a solid or powdered sample [2,13,15].

Significant progress has been made to optimize the extraction and the potential use of valuable compounds from olive leaves. However, the impact of post-harvest conditions on the chemical composition of extracts is not yet fully understood, given that many bioactive compounds are heat-sensitive and prone to degradation. Additionally, olive pruning biomass is generated in large quantities over a short period, typically one to two months, and has a high water content ranging between 43% and 51%, which renders it susceptible to physico-chemical and biological deterioration reactions [3,9,16]. As a result, olive leaves are perishable and can only be stored in a fresh state for a few days. Hence, reducing the moisture content of olive leaves during storage prior to processing can help preserve their nutritional and functional components [10]. Currently, drying is the most studied method for addressing this issue [17,18,19]. However, selecting an appropriate drying method is of utmost importance, because, while drying is known to inhibit microbial/enzymatic activity and to attenuate the reactions of oxidation, this thermal treatment could modify the shape, the texture, the nutritional value and the chemical composition of the bioactive fraction of plant materials [17,19,20,21].

Although the effects of drying on the nutritional and functional value of olive leaves are not yet fully understood, several studies have been carried out in recent years paying particular attention to some techniques such as air-drying, oven-drying, infrared-drying, radio frequency-drying, microwave-drying, spray-drying, freeze-drying and solar-drying [13,22]. The air-drying technique was proposed to be the most effective method for drying olive leaves from Spanish cultivars. This technique resulted in maintaining high levels of bioactive compounds, such as oleuropein, and improved the antioxidant activity of the resulting extracts [23].

Feng et al. also reported that ambient-air-drying resulted in the highest content of phenolic compounds [18], and did not have any adverse effects on the nutritional value of olive leaves, making it an effective and simple drying method for their preservation [19]. However, another study found that microwave- and freeze-drying had better results compared to air-drying and other techniques, with higher phenolic compound content and antioxidant power [20]. Additionally, drying olive leaves at 120 °C also induced stronger antioxidant activity and a higher content of phenolic compounds, mainly secoiridoid derivatives [24]. Regardless of the drying method used, however, it is generally known that high temperatures and long drying times can destroy or alter the structure of some bioactive compounds [2].

The selection of an appropriate air circulation system is important for ensuring a constant air flow that enables an efficient and uniform reduction in the moisture content in olive leaves during the drying process [2].

Finally, in spite of numerous studies, it is worth highlighting the insufficient information and research dedicated to evaluating how various drying methods affect the bioactive and nutrient compound content of leaves of various varieties cultivated in specific olive oil-producing countries. Morocco stands out as a stark example of this gap, with almost no research addressing this issue, impeding the development of valorization strategies.

This study therefore aimed to initiate a procedure for valorizing olive leaves resulting from the pruning of olive groves in the Meknès El Hajeb region, one of the main olive-growing regions of Morocco, with a view to extracting valuable ingredients from these leaves. Considering that the preservation of this raw material until its processing is crucial for the success of the planned valorization strategy, the focus of this study was to investigate the effects of four drying methods (air-drying, oven-drying, solar-drying and freeze-drying) on the protein content, total phenolic content, flavonoid richness and concentration of individual phenolic compounds in olive leaves of the main olive cultivars, i.e., ”Picholine Marocaine”, “Menara”, “Arbequina” and “Koroneiki”, cultivated in Morocco.

## 2. Results and Discussion

### 2.1. The Effect of ‘Variety’ and ‘Drying Method’ on the Nutritional and Bioactive Content of Olive Leaves

A two-factor analysis of variance (ANOVA 2) was conducted to test the effect of ‘variety’ and ‘drying method’, as well as their interaction, on the nutritional value (total nitrogen and crude protein content) and bioactive compound content (total polyphenol and total flavonoid content). The results are presented in Table S1 in the Supplementary Materials.

The obtained results demonstrate a significant impact (*p* < 0.05) of both the ‘variety’ and ‘drying method’ factors, as well as their interaction, on the nutritional and bioactive compound contents of olive leaves. This finding is consistent with the research conducted by Filgueira-Garro et al., 2022, who observed similar significant effects of different cultivars and drying methods (vacuum-drying, oven-drying, freeze-drying and air-drying) on the total phenolic content and concentration of specific phenolic compounds such as hydroxytyrosol and oleuropein [23]. The harvesting period of olive leaves was also reported to be critical for obtaining higher bioactive compound content. Leaves collected during November and dried exhibited higher levels of phenolic compounds, mainly secoiridoids and their derivatives, compared to leaves collected during other periods [25].

### 2.2. Effect of the Drying Process on the Total Nitrogen and Crude Protein Contents of Olive Leaves

The contents of nitrogen and crude protein were initially determined in fresh leaf samples. The results are presented in Table 1, showing the average values for these measurements (as a percentage of fresh weight (%)).

Based on these results, it can be inferred that the total nitrogen content in the studied samples exhibited a range of 0.76% to 0.98%, while the crude protein content showed variation between 4.78% and 6.11%. Notably, the “Menara” cultivar exhibited the lowest concentration for both parameters compared to the other cultivars. Among the studied cultivars, “Picholine Marocaine” displayed the highest content for both total nitrogen (0.98%) and crude protein (6.11%) content. This was followed by the “Koroneiki” and the “Arbequina” cultivars, although no statistical difference was found between the three cultivars.

The analysis of variance test results for nitrogen and crude protein content data (Table 1) indicate that there are no statistically significant differences among the leaf samples of the three cultivars “Arbequina”, “Picholine Marocaine” and “Koroneiki”. However, a significant difference was observed when comparing the content of these compounds in the leaves of these three varieties with the “Menara” cultivar samples. These findings are consistent with previous studies investigating the nutritional composition of different olive cultivars [10,26,27]. These results emphasize the significant variations in total nitrogen and crude protein contents observed among the analyzed cultivars, highlighting the importance of selecting suitable varieties to optimize the nutritional value of olive leaves.

**Figure 1:**
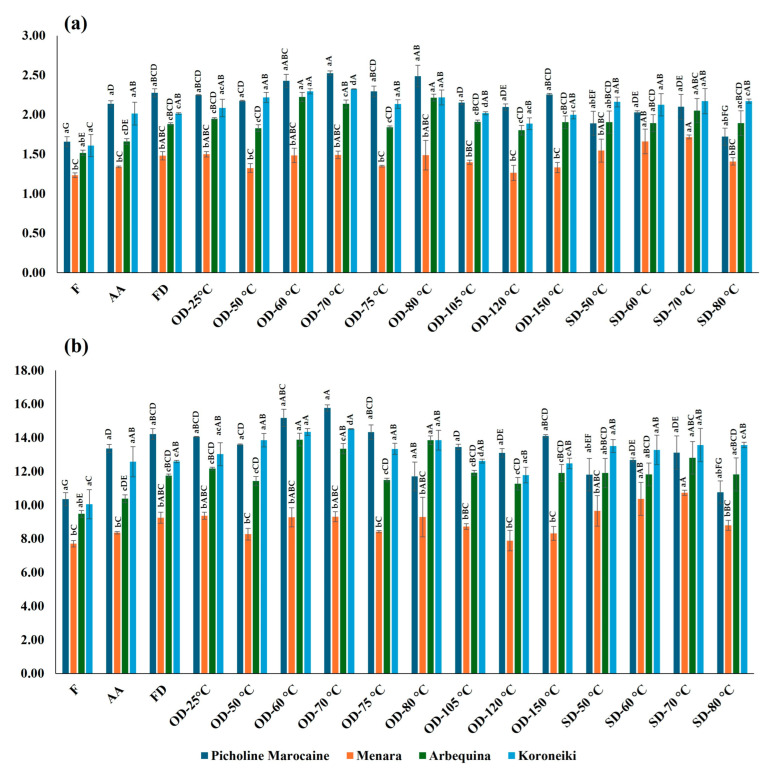
Mean values and standard deviation for (**a**) total nitrogen and (**b**) crude protein content in fresh (% of EDW) and dried leaves (% of DW) in the analyzed samples according to variety and drying method applied. Bars with different capital letters represent significant differences between the drying methods for the same variety, while bars with different lower-case letters show significant differences between the varieties for the same drying method (*p* < 0.05) (Student–Newman–Keuls (SNK) test). Abbreviations: AA: ambient-air-drying; F: fresh; FD: freeze-drying; OD: oven-drying; SD: solar-drying.

Additionally, as mentioned earlier, to better evaluate the impact of the investigated drying methods on the initial nitrogen and total protein contents in fresh leaves, the results were expressed as a percentage relative to the dry weight (%). This enables us to compare the concentration of these components in the dry matter of fresh leaves with that in dried leaves. Lastly, the outcomes derived from this data conversion underwent a comparative statistical analysis alongside the other findings resulting from the application of the assessed drying methods in this study. The results are depicted in Figure 1a,b.

In the case of the “Arbequina” cultivar, it was observed that the drying method had a significant effect on the content of total nitrogen and crude proteins. Specifically, the leaves oven-dried at 60 °C and 80 °C showed the highest content of total nitrogen and crude protein. On the other hand, ambient air-dried leaves showed the lowest contents of total nitrogen and crude protein, with mean values of 1.66% and 10.38%, respectively. These findings indicate that oven-drying at 60 °C or 80 °C is the most suitable method for preserving the nitrogen and protein content of “Arbequina” leaves as compared to the ambient-air-drying method.

For the “Koroneiki” leaf samples, the highest content was observed in the leaves oven-dried at 70 °C (2.32% and 14.52%, respectively), followed by the samples oven-dried at 60 °C, while oven-drying at 120 °C showed the lowest total nitrogen and crude protein contents. This variation in total nitrogen and crude protein indicates that the drying method has a significant effect on the nutritional content of “Koroneiki” leaves.

In the case of “Menara” leaves, the samples dried using the solar-drying method at 70 °C demonstrated the highest contents (1.72% and 10.74%, respectively), followed by those solar-dried at 60 °C, while the leaves dried in the oven at 120 °C were associated with the lowest content.

Among the drying methods evaluated, oven-drying at 70 °C was found to be the most effective for “Picholine Marocaine” leaves, exhibiting the highest total nitrogen and crude protein contents of 2.52% and 15.77%, respectively. This was closely followed by oven-drying at 80 °C, while the leaves solar-dried at 80 °C displayed the lowest contents of total nitrogen and crude protein.

Moreover, the results indicate that the total nitrogen and crude protein contents increased in the dried samples compared to the fresh leaves. A higher drying temperature has been shown to induce a decrease in protein content, whereas mild drying temperatures ranging between 30 °C and 60 °C in an oxygen-free environment have been found to preserve protein and other heat-sensitive nutrients [22]. In a study conducted by Alibas and co-workers on apple peel, drying exhibited a significant increase in the total nitrogen amount of dried samples compared to fresh ones [28]. The dehydration of drumstick leaves was also associated with a statistically significant increase in total protein content comparing to fresh samples [29]. In another report, Mansour et al. (2016) observed that drying moringa leaves resulted in an increase in protein content [30].

### 2.3. Effect of the Drying Process on Total Phenolic Content

The total phenolic content of the fresh leaves, expressed as gallic acid equivalents per fresh weight (GAE/g FW), from the four cultivars under study ranged from 9.02 to 20.18 mg GAE/g FW (Table 2). The highest content was observed in “Arbequina” leaves, followed by “Menara” leaves and “Koroneiki” leaves. In contrast, “Picholine Marocaine” samples exhibited the lowest level of polyphenols. These findings are consistent with previous studies conducted on other olive tree cultivars [10,31].

The statistical significance of these differences was assessed using the ANOVA test. The results shown in Table 2 clearly indicate that the polyphenol contents of the “Koroneiki” and “Menara” varieties were comparable, with no significant differences. However, the other two varieties exhibited significant distinctions. “Arbequina” stood out with a notable polyphenol content, while “Picholine Marocaine” appeared to have lower levels of these compounds compared to the other varieties. The observed differences for these two varieties were statistically significant.

When expressed as equivalent dry weight (EDW) (Table 3), the total phenolic content of the fresh leaves from the four studied varieties exhibits a similar trend, ranging from 15.46 to 34.94 mg GAE/g EDW. Specifically, the highest content was found in “Arbequina” leaves, followed by “Koroneiki” samples and “Menara” leaves. “Picholine Marocaine” samples showed the lowest total phenolic content.

These outcomes are in line with previous studies investigating the total phenolic content of olive leaves from other cultivars. For example, Filgueira-Garro et al. (2022) found that fresh conventional and ecological “Arbequina” leaves induced an average total phenolic content of 15.0 and 24.1 mg GAE/g of dry weight (DW), respectively [23]; the reported values for the variety “Chétoui” are relatively similar, with concentrations fluctuating between 16.5 and 24.9 mg GAE/g DW [32], while for 15 Italian cultivars, the total phenolic content ranged between 11 and 49 mg GAE/g DW [33].

The phenolic content of the four studied cultivars after drying is shown in Table 3. “Arbequina” leaves oven-dried at 25 °C exhibited the highest content, followed by ambient air-dried leaves. Meanwhile, leaves solar-dried at 50 °C were associated with the lowest content. The “Koroneiki” leaves dried in ambient air induced the highest content, followed by the samples dried in the oven at 25 °C. The lowest content was found in the “Koroneiki” leaves dried by the solar dryer at 50 °C.

In the case of “Picholine Marocaine”, the leaves dried in ambient air exhibited the highest content, followed by the leaves dried in the oven at 25 °C. “Picholine Marocaine” leaves dried by the solar dryer at 50 °C were found to be the poorest in terms of total polyphenols. Likewise, air-dried “Menara” leaves presented the highest phenolic content, followed by the freeze-dried samples. However, the lowest content was recorded in the “Menara” samples dried by the solar dryer at 50 °C.

Higher total phenolic contents in dried leaves compared to those in a fresh state was also reported by Boudhrioua et al., 2009 [10]. The impact of higher temperatures may be attributed to the increased solubility of phenolic compounds. Higher temperatures tend to break cellular structures and thus facilitate the release of phenolic compounds [34]. Another similar explanation suggests that drying may lead to the destruction of cell walls and enhance the liberation of polyphenols [10]. Drying may also enhance the penetration of extraction solvents into the plant matrix by damaging cell structures [25]. It has been shown that solvents can more easily enter cells with a smaller surface area, as demonstrated in prior studies [35].

Other studies have shown a decrease in the antioxidant capacity and total phenolic content of dried olive leaves, regardless of the drying method [36]. This decrease may be due to the lower protection of phenolic compounds in extracts compared to raw materials during the drying process. Similar results were reported for the drying of bayberry juice as the total phenolic content decreased by 4% in the dried extracts [37]. Similarly, other authors have reported a significant loss in the contents of individual phenolic compounds in dried cherry fruits [38].

The results obtained from applying these drying methods to the studied leaves are consistent with the literature, which indicates that drying leaves at room temperature leads to a high phenolic content [18]. Similar outcomes were reported by Filgueira-Garro et al. (2022), as air-drying induced higher antioxidant capacity and phenolic compound content, mainly oleuropein [23]. A moderate air-drying temperature is also believed to be responsible for the preservation of phenolic compounds [23].

The high content of total polyphenols in freeze-dried “Menara” leaves is likely due to the formation of ice crystals in the plant matrix, which causes the disruption of plant tissues, resulting in better diffusion of phenolic compounds to the solvent [20]. Previous studies have shown that when compared to air-drying, freeze-drying and microwave-drying are more effective in producing higher levels of phenolic compounds from olive leaves [20]. The convective drying of Brazilian “Arbequina” olive leaves at a temperature of 70 °C also induced better results at 50 °C in terms of phenolic content [39]. Similarly, drying olive leaves at higher temperatures (120 °C) led to increased levels of secoiridoids, flavonoids, triterpenoids and other polar compounds [25]. However, it has been demonstrated that every phenolic compound responds to drying temperatures in a different way [40]. Other authors have supported this tendency, revealing that while certain phenolic compounds’ content increased linearly as the drying temperature rose, others decreased and were undetectable at higher temperatures [41,42].

### 2.4. Effect of the Drying Process on Total Flavonoid Content

The total flavonoid content in the fresh olive leaves of the studied cultivars (expressed as quercitrin equivalents (QE)/g of FW) ranged from 2.75 to 3.28 mg QE/g FW (Table 2). While there were no significant differences among the four varieties, both “Menara” and “Koroneiki” leaves showed the highest content in the fresh olive leaves, followed by “Arbequina”, while “Picholine Marocaine” leaves had the lowest total flavonoid content.

When the total flavonoid content of the fresh olive leaves was expressed as QE per g of EDW, the concentrations ranged from 4.67 to 5.75 mg QE/g EDW (Table 4). The “Koroneiki” leaves exhibited the highest content, followed by the “Arbequina” and “Menara” leaf samples, while “Picholine Marocaine” leaves had the lowest total flavonoid content. Similar findings were reported by Lorini and colleagues, with flavonoid content ranging from 3.94 to 5.94 mg QE/g DW in olive leaves of the “Arbequina”, “Manzanilla” and “Picual” cultivars collected during different seasons of the year (autumn, winter, spring and summer) [43].

The total flavonoid contents of the fresh samples and those dried by the four drying methods are presented in Table 4. In the case of “Arbequina”, the leaf samples dried in ambient air recorded the highest total flavonoid content, followed by the leaves oven-dried at 25 °C. The lowest content was observed in the leaves solar-dried at 50 °C. Likewise, the “Koroneiki” leaves dried by means of ambient-air-drying presented the highest content, followed by those dried in the oven at 25 °C, while drying the “Koroneiki” leaves with the solar dryer at 50 °C gave the lowest value.

The “Picholine Marocaine” samples showed the highest total flavonoid content in leaves dried at room temperature, followed by those dried in an oven at 150 °C. Leaves dried using a solar dryer at 70 °C showed the lowest content. The highest flavonoid content was observed in the freeze-dried “Menara” leaves, followed by air-dried leaves. The lowest content was found in the leaves dried by the solar dryer at 70 °C.

The observed rise in the total flavonoid content in the dried leaves compared to the fresh samples can be attributed to the potential binding of flavonoids with other compounds or to structural changes occurring in the flavonoids themselves [44]. Other researchers have also related this increase to some biochemical reactions that occurred during the drying process [45]. These outcomes are consistent with those reported by Ghelichkhani et al., 2019, by using spray- and freeze-drying [26]. Drying onion slices by means of sun-drying, oven-drying and microwave-drying also induced the highest phenolic contents when compared with fresh samples [46]. Similar results were also observed when drying tomatoes by means of freeze- and hot-air-drying [44].

The highest total flavonoid content in ambient air-dried extracts may be related to the inhibition of oxidative and hydrolytic enzymes, which prevented the loss of these compounds [45]. Air-drying at room temperature also induced higher flavonoid content compared to drying at higher temperatures and freeze-drying in a study conducted by Feng et al., 2021 [18].

Although ambient-air-drying is associated with the highest total flavonoid content, a high drying temperature during a short processing time may induce a reduction in flavonoid compound losses according to previous work published by Erbay and Icier, (2009) [46]. A similar tendency was observed by Kamran et al., 2015 [16]. However, although it has been demonstrated that these compounds behave differently towards drying temperatures, as already mentioned, high drying temperatures may tend to diminish flavonoid content [25]. Another study revealed a higher efficiency of microwave-drying, freeze-drying, vacuum-drying and oven-drying when compared with ambient-air-drying for obtaining a dried olive leaf extract rich in flavonoids [20]. Microwave-drying was also reported to be a more efficient method for obtaining more polyphenol-rich dried extracts in a study conducted by Arslan and Özcan, (2010) [47].

### 2.5. Effect of Drying on Olive Leaves’ Bioactive Fraction

Figure S1 in the Supplementary Materials depicts representative chromatograms obtained by HPLC-DAD analysis of fresh and dried samples of the “Picholine Marocaine” variety predominant in Morocco.

**Figure 2:**
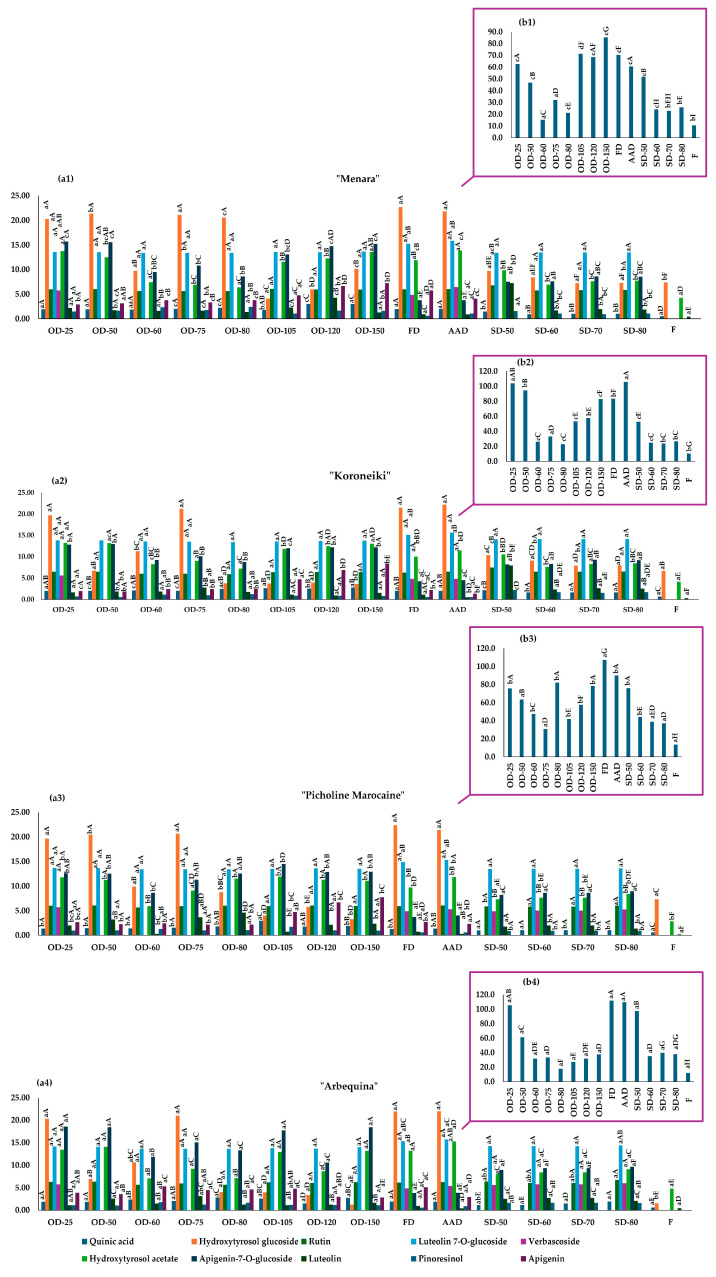
Concentration of phenolic compounds identified in the fresh (expressed as mg/kg EDW) and dried samples (expressed as mg/kg DW): (**a**) content of quinic acid, hydroxytyrosol glucoside, rutin, luteolin 7-*O*-glucoside, verbascoside, hydroxytyrosol acetate, apigenin 7-*O*-glucoside, luteolin, pinoresinol and apigenin; (**b**) content of oleuropein. Bars with different capital letters represent significant differences between the drying methods for the same variety, while bars with different lower-case letters show significant differences between the varieties for the same drying method (*p* < 0.05). Abbreviations: AA: ambient-air-drying; F: fresh; FD: freeze-drying; OD: oven-drying; SD: solar-drying.

The bioactive fraction of the analyzed samples was first determined in their fresh state. Five phenolic compounds were identified and quantified in the fresh olive leaves, including quinic acid, hydroxytyrosol glucoside, hydroxytyrosol acetate, oleuropein and luteolin. Oleuropein was the most abundant phenolic compound in all varieties analyzed and varied between 16.84 and 23.02 mg/kg FW for the “Menara” and “Picholine Marocaine” samples (Figure S2 in Supplementary Material). For reasons of clarity, the results of the oleuropein concentration in Figure S2 and Figure 2 are presented on a different scale than those of the other phenolic compounds, which have a relatively narrow range of values. Hydroxytyrosol glucoside was the second most abundant phenolic compound in “Picholine Marocaine”, “Menara” and “Koroneiki” (12.38, 11.90 and 11.74 mg/kg FW, respectively), while the samples of ‘Arbequina’ had the lowest content (2.70 mg/kg FW). For hydroxytyrosol acetate, the fresh leaves of ‘Arbequina’ were the richest (8.34 mg/kg FW), followed by “Koroneiki” (7.32 mg/kg FW), then “Menara” (6.86 mg/kg FW) and finally “Picholine Marocaine” (4.85 mg/kg FW). The lowest concentrations of the detected compounds were found in quinic acid (between 0.81 and 1.19 mg/kg FW for the leaves of “Menara” and “Koroneiki”, respectively) and luteolin (between 0.29 and 0.80 mg/kg FW for the samples of “Picholine Marocaine” and “Arbequina”, respectively).

The content of oleuropein, expressed as EDW, ranged between 10.28 and 13.58 mg/kg EDW in the samples of “Koroneiki” and “Picholine Marocaine”, respectively. The lowest content of hydroxytyrosol glucoside was found in the sample of “Arbequina”, while the leaves of “Menara” had the highest concentration (1.56 and 7.38 mg/kg EDW, respectively). The concentrations of hydroxytyrosol acetate ranged from 2.86 to 4.84 mg/kg EDW in the samples of “Picholine Marocaine” and “Arbequina”. Finally, the highest content of quinic acid was found in the leaves of “Koroneiki” (0.68 mg/kg EDW) and the lowest in “Menara” (0.50 mg/kg EDW), while the contents of luteolin in the samples of “Picholine Marocaine” and “Arbequina” ranged from 0.17 to 0.46 mg/kg EDW.

Analyzing the dried samples, eleven phenolic compounds were identified and quantified, namely quinic acid, hydroxytyrosol glucoside, rutin, luteolin 7-*O*-glucoside, verbascoside, hydroxytyrosol acetate, apigenin-7-*O*-glucoside, oleuropein, luteolin, pinoresinol and apigenin. The observed increase in the number and content of phenolic compounds identified in the dried samples suggests that drying may lead to the destruction of cell walls and the subsequent release of phenolic compounds [10]. Another possible explanation could be the increased penetration of extraction solvents into dried matrices, which could result from damage to the cell structures [25]. This result is consistent with other studies indicating that the content of certain phenolic compounds in fresh olive leaves was below the limit of quantification, while it increased in dried samples [23,48].

Like in fresh leaves, oleuropein was the most abundant phenolic compound in all dried samples, with concentrations ranging from 15.24 to 111.82 mg/kg DW for the “Menara” samples oven-dried at 60 °C and the freeze-dried “Arbequina” samples, respectively (Figure 2). It is noteworthy that the enzymatic activity of polyphenol oxidase may contribute to the degradation of oleuropein in dried samples; however, in fresh samples, both β-glucosidase and polyphenol oxidase are likely involved [49]. Freeze-dried samples had the highest oleuropein content compared to the other methods analyzed (111.82 and 107.14 mg/kg DW for “Arbequina” and “Picholine Marocaine”, respectively), except for the “Koroneiki” and “Menara” samples, which had higher oleuropein content when air-dried (105.48 mg/kg DW) and oven-dried at 150 °C (85.30 mg/kg DW), respectively. The high oleuropein content in all analyzed varieties is consistent with the literature [11,16,17,50]. It has been demonstrated that freeze-drying and air-drying can lead to an increase in the concentration of phenolic compounds, including oleuropein and hydroxytyrosol, in different cultivars [23,48]. Other authors have also reported that the concentration of the main phenolic compounds increased with freeze-drying and hot-air-drying at temperatures up to 120 °C [17]. Zhang et al. (2022) found that hot-air-drying can lead to higher contents of oleuropein, while freeze-drying increased the content of flavonoids such as luteolin and apigenin regardless of the cultivar [48].

If we focus on oven-drying at different temperatures, no clear trend with increasing temperature was observed. For example, for quinic acid and apigenin, the highest contents in the oven-dried samples were found at higher drying temperatures (105 to 150 °C) for all varieties analyzed, with the exception of the “Arbequina” samples, which showed a higher concentration of these compounds at temperatures between 60 and 105 °C. Low drying temperatures led to higher concentrations of rutin, hydroxytyrosol acetate, apigenin 7-*O*-glucoside and, in the samples of “Picholine Marocaine” and “Arbequina”, luteolin 7-*O*-glucoside and oleuropein in all varieties. The highest levels of the latter two phenolic compounds were found at 150 °C for the samples “Koroneiki” and “Menara”. Feng et al. (2021) also reported a decrease in the content of the potent phenolic compounds, namely oleuropein and hydroxytyrosol, in olive leaves with an increase in oven-drying temperature from 25 to 70 °C [18]. Verbascoside was not detected in the samples oven-dried at temperatures above 25 °C for any of the varieties analyzed, suggesting that it is susceptible to the effects of high temperatures. However, Ahmad-Qasem and colleagues revealed a statistically significant increase in the content of verbascoside when increasing the temperature of hot-air-drying from 70 to 120 °C [17]. In the case of the solar-dried “Picholine Marocaine” and “Arbequina” samples, it was observed that the content of verbascoside increased linearly with increasing temperature, from 50 up to 80 °C. This suggests that each cultivar may respond differently to different drying conditions.

The results obtained also showed that solar-drying at 50 °C was associated with a higher content of oleuropein than the other higher temperatures of solar-drying. This suggests that the degradation of oleuropein may occur at higher temperatures during the solar-drying process. Both hydroxytyrosol glucoside and apigenin could not be detected in the solar-dried samples of the varieties “Picholine Marocaine” and “Arbequina”, while verbascoside and apigenin could not be detected in “Koroneiki” and “Menara”. In addition, solar-drying at 50 °C showed higher rutin contents compared to all other drying methods. This could be due to the possible correlation between the genetically determined response of each olive variety to stress conditions and the subsequent degradation of these compounds during the drying process.

Finally, it was found that the ambient air-dried samples were richest in hydroxytyrosol glucoside, luteolin 7-*O*-glucoside and hydroxytyrosol acetate in the “Arbequina” variety, in rutin and luteolin 7-*O*-glucoside for the variety “Picholine Marocaine”, in luteolin 7-*O*-glucoside, verbascoside and hydroxytyrosol acetate for “Menara”, and in hydroxytyrosol glucoside, luteolin 7-*O*-glucoside and oleuropein for “Koroneiki”. Şahin et al. (2017) reported that ambient-air-drying was a less effective drying method compared to microwave-, freeze-, vacuum-, and oven-drying [20]. Andrejč et al. (2022) found that the ambient-air-drying of olive leaf samples induced higher levels of oleuropein and other phenolic compounds compared to air-drying at 105 °C, while freeze-drying was the most efficient of all the techniques studied [51].

It can be concluded that, of the methods considered in this work, ambient-air-drying and freeze-drying seem to be the most suitable. However, their industrial application may prove challenging, and further economic studies are therefore required to identify their cost-effectiveness. It is evident that the economic viability of freeze-drying on a large scale is questionable, particularly in view of the considerable energy consumption involved. Additionally, industrial-scale freeze-dryers present significant challenges due to the necessity of maintaining optimal drying conditions, ensuring a uniform drying process, and implementing real-time controlling and monitoring systems while adhering to national and international regulatory standards.

When focusing on the ambient-air-drying technique, its primary drawbacks are the necessity for extensive space to enhance the interaction between the biomass and atmospheric air, as well as the prolonged drying time. In comparison to other drying technologies, this technique might be less economically viable. Therefore, methods such as solar-drying appear to have considerable potential, particularly given their use of a renewable energy source: solar energy. Further research is required to develop and optimize renewable-energy-based technologies for the drying of the residual biomass derived from olive grove pruning.

## 3. Materials and Methods

### 3.1. Chemicals and Reagents

Folin–Ciocalteu reagent, sodium carbonate (Na_2_CO_3_), sulfuric acid, sodium nitrite (NaNO_2_), aluminum chloride (AlCl_3_) and sodium hydroxide (NaOH) were purchased from Sigma-Aldrich (St. Louis, MO, USA). Double deionized water with a conductivity of 18.2 MΩ was prepared in the laboratory using a Milli-Q system (Millipore, Bedford, MA, USA). In the case of HPLC analysis, all solvents used were of HPLC-grade purity and were used without further purification. Acetonitrile and acetic acid for the preparation of the mobile phase were purchased from Sigma-Aldrich (St. Louis, MO, USA), while ethanol for the preparation of the standard solutions was supplied by Prolabo (Paris, France). Commercial pure standards of apigenin, caffeic acid, ferulic acid, hydroxytyrosol, gallic acid, luteolin, *p*-coumaric acid, pinoresinol, quinic acid, tyrosol and rutin were obtained from Sigma-Aldrich (St. Louis, MO, USA). The standards for apigenin 7-*O*-glucoside, luteolin 7-*O*-glucoside, oleuropein and verbascoside were obtained from Extrasynthese (Lyon, France).

### 3.2. Sampling

During the olive tree pruning period of the 2021/2022 crop season, samples of olive leaves were collected from four olive orchards located in the Meknes-El Hajeb region of Morocco. The first two orchards were twenty-year-old high-density irrigated orchards, with a tree density of 1250 trees per hectare. One of these orchards was planted with “Arbequina” *cv.* olive trees, while the other featured “Koroneiki” *cv.* olive trees. The third orchard consisted of a traditional “Picholine Marocaine” *cv.* grove, boasting an age of over 50 years and a tree density of 100 trees per hectare. This grove was managed under an irrigation system. Lastly, the fourth sampling orchard was a 9-year-old rainfall “Menara” olive tree orchard, planted at a density of 285 trees per hectare.

The selection of olive cultivars and growing systems for this study was based on their common usage in Morocco. “Picholine Marocaine” is the most widely cultivated variety in Morocco. Although non-irrigated farming systems are commonly adopted nationwide, including in the Meknes-El Hajeb region, irrigated orchards are becoming increasingly popular throughout the country. Additionally, high-density planting systems are prevalent in the Meknes-El Hajeb region and other olive-growing regions in Morocco, with “Arbequina” and “Koroneiki” being the main cultivars used in this type of planting system.

A systematic sampling approach was employed to collect olive leaf samples from each of the four studied cultivars. Two samples, weighing two kilograms each, were collected from pruning twigs obtained from randomly selected trees in each orchard. To ensure representative sampling, the twigs were collected from the four cardinal points of the selected trees. Upon collection, the leaves were carefully separated from the twigs and branches. They were then placed in individual bags to maintain their freshness and transported to the laboratory within two hours of collection to minimize any potential degradation. At the laboratory, each of the two samples from each cultivar was then coded and divided into 16 batches of 50 g and allocated to the investigated drying techniques: one batch for fresh samples, one for ambient-air-drying, nine for oven-drying, four for solar-drying, and one for freeze-drying. In total, 128 samples were included in this study, allowing for a comprehensive analysis and comparison of the different drying techniques and their effects on the olive leaves’ health-related compound content.

### 3.3. Drying Methods

As there were no previous studies on the drying of the leaves of olive varieties grown in Morocco, the choice of drying methods was based on conclusive research results obtained in other olive-growing countries in the Mediterranean region. According to the available scientific literature [9,10,16,17,20,29], air-drying, oven-drying, sun-drying and freeze-drying were selected for the evaluation. By using these widely recommended methods, we aimed to ensure consistency and comparability with previous research findings.

Before drying the samples, their initial moisture content was determined by drying a certain amount of each sample in an oven set at 105 °C until a constant weight was reached (approximately 48 h). The difference in weight before and after oven-drying gave the moisture content of the sample [47].

#### 3.3.1. Air-Drying

The samples were laid on a bench to dry. The leaves were exposed to indirect sunlight coming from the windows, as well as regular laboratory lighting. Continuous ventilation was ensured by keeping the windows open throughout the drying process. The laboratory temperature fluctuated between 22 °C and 28 °C. Every 3 days, the samples were weighed to monitor water evaporation.

#### 3.3.2. Oven-Drying

Nine different temperatures (25, 50, 60, 70, 75, 80, 105, 120 and 150 °C) were tested to dry the samples in the oven at different times. The drying time for each of these temperatures was determined by plotting the evolution of the evaporation of the contained water in the studied samples as a function of time (Figure S3 in Supplementary Materials).

The drying time, which gives the less constant weight, was determined for each temperature, and then applied for drying the samples. As depicted in Figure S3, 72 h was found to be effective for drying at a temperature of 25 °C, 560 min at a temperature of 50 °C, 240 min at a temperature of 60 °C, 120 min at a temperature of 70 °C, 110 min at a temperature of 75 °C, 70 min at a temperature of 80 °C, 50 min at a temperature of 105 °C, 35 min at a temperature of 120 °C and 25 min at a temperature of 150 °C.

#### 3.3.3. Freeze-Drying

The samples were freeze-dried in a freeze-dryer (Ilshin Lab. Co., Ltd., Yangju-si, Republic of Korea). The drying time was controlled to obtain a freeze-dried product (samples were placed in vials, connected to an aluminum tray, frozen at −80 °C and then transferred to the freeze-drying chamber where the shelves were pre-cooled to −50 °C). The temperature of the heat transfer fluid was maintained at −50 °C for 36 h. The samples were immediately sealed and vacuum packed in plastic bags. Finally, the samples were stored at room temperature.

#### 3.3.4. Solar-Drying

The solar-drying of olive leaves was carried out using an indirect, partially solar, modular dryer with forced convection and an electrical backup. The experiments were conducted with a sample size of 50 g at drying air temperatures of 50, 60, 70 and 80 °C and at an air flow rate of 300 m^3^/h. After 30 min of idle operation, the leaves were spread on the first rack of the dryer, and the temperature was set using a control box. The air flow was controlled by a fan with two levels of flow (150 and 300 m^3^/h). The wet matter content Mh(t) of the product was determined by static weighing every 5 min at the beginning of the tests and up to 20 min towards the end. The drying times for each temperature were determined as follow: 260 min for 50 °C; 180 min for 60 °C; 120 min for 70 °C; and 70 min for 80 °C. Vacuum packing using polyethylene (PE) bags was employed to store the dried olive leaves at room temperature.

### 3.4. Grinding, Sieving and Storing Samples

Once the desired level of dryness was attained, the samples dried using each respective method were ground on the same day using a BLET knife mill (BLET Measurement Group, Paris, France) and then sieved through a sieve with a particle size of 250 µm. The samples were then placed in hermetically sealed plastic bags and stored at −45 °C until analysis.

### 3.5. Nutritional and Bioactive Fraction Analysis

To investigate the nutritional fraction and bioactive compounds of the obtained samples, triplicate extractions were performed for each sample. Subsequently, each extract underwent three separate analyses, ensuring the utmost accuracy and reproducibility in this study.

To achieve the main objective of the present work, we proceeded as follows: Initially, we determined the content of these compounds in the fresh leaf samples and expressed it relative to the FW. However, to facilitate the comparison of these results with those obtained after drying the samples, it was necessary to convert the content and express it in terms of an EDW. This conversion was performed by using the moisture content of each sample to convert the compound content, initially expressed relative to the fresh weight, into an equivalent concentration based on the dry weight. This approach allowed us to represent the content of these compounds in fresh leaves on a dry weight basis, ensuring accurate and meaningful comparisons.

Thus, to convert the content from mg/g FW to mg/g EDW, the following equation was utilized:

(1) $$\mathrm{Content}\;\mathrm{in}\;\mathrm{mg}/g\;\mathrm{EDW}\;=\frac{\left(\mathrm{Content}\;\mathrm{in}\;\mathrm{mg}/g\;\mathrm{FW}\right)\;}{\left(1-\;W\right)}$$

Here, W represents the moisture content of the sample, expressed as a fraction.

#### 3.5.1. Total Nitrogen and Crude Protein Content

This content was determined by the “KJELDAHL” method in three steps according to the AFNOR T90-110 standard (1983) [52,53]. The analysis was performed three times for each sample. The nitrogen content was determined using sulfuric acid with a normality of 0.1 N, and the crude protein content was determined using the conversion factor 6.25.

#### 3.5.2. Total Phenolic Content

First, the phenolic fraction was extracted by using the procedure previously reported by Olmo-García and colleagues [54,55]. Briefly, sample extracts were prepared by mixing 0.1 g of each sample with 10 mL of EtOH/water (60:40, *v*/*v*). Afterwards, the mixture was shaken for 3 min in a vortex, and then was put into an ultrasound bath (Bandelin, SONOREX DIGIPLUS, Berlin, Germany) for 30 min and centrifuged for 15 min at 5000 rpm. The supernatant was recovered in a flask and the same procedure was repeated by adding 10 mL of EtOH/water (80:20, *v*/*v*). Once the supernatant was collected, the procedure was repeated one last time by adding 100% of ethanol. All the supernatants coming from the 3 extraction cycles were mixed, and around 2 mL of the obtained extract was filtered by using a Clarinert^®^ nylon syringe filter of 0.22 µm (Agela Technologies, Torrance, CA, USA) and then stored at a temperature of −45 °C until analysis.

Afterwards, the determination of total phenols was performed according to the Folin–Ciocalteu method modified according to Bajoub et al., 2014 [56]. Briefly, 1 to 5 mL of the three combined extracts was diluted in 35 mL of distilled water, and then, 2.5 mL of the Folin–Ciocalteu reagent was added. Subsequently, 5 mL of Na_2_CO_3_ solution (6%) was introduced, while distilled water was gradually poured in until the total volume reached 50 mL. The solution was homogenized and left under dark conditions for 30 min, and the absorbance was evaluated at 760 nm using a Varian Cary 50 UV–Vis Spectrophotometer. A calibration curve was prepared using gallic acid as a standard. The results were expressed as mg GAE/g of FW or DW or EDW.

#### 3.5.3. Total Flavonoid Content Determination

A total of 1000 µL of the olive leaf extract was mixed with 300 µL of a 7% NaNO_2_ solution, and then, the mixture was incubated for a few minutes at room temperature. Afterwards, 600 µL of a freshly prepared 10% AlCl_3_ solution was added to the mixture before completing it with 2000 µL of NaOH (1 M). The final volume was adjusted to 10 mL with distilled water and the absorbance of this solution was measured at 510 nm a Varian Cary 50 UV–Vis Spectrophotometer (Santa Clara, CA, USA). A Calibration curve was prepared by using quercitrin as a pure standard, and the obtained results were expressed as QE/g of FW or DW or EDW.

#### 3.5.4. Liquid Chromatographic Analysis of Bioactive Fraction

From each sample analyzed, 1 mL aliquots of the phenolic extract, previously used to determine the total phenolic content of the sample, were transferred to amber glass vials specifically designed for analysis by HPLC.

The chromatographic analysis of the phenolic compounds in the olive leaf samples was carried out using an HPLC system, specifically the Agilent 1260 Infinity II LC System (Agilent, Santa Clara, CA, USA). This instrument is equipped with a quaternary pump (model G7111B), a vacuum degasser, an autosampler (model G7129A), a thermostatic column compartment (model G7130A) and a diode array detector (DAD, model G7115A). Instrument control, data acquisition and processing were carried out using OpenLAB CDS ChemStation Edition software (Version 3.6(3.6.0), Agilent Technologies, Santa Clara, CA, USA).

A mobile phase consisting of acidified water (1% acetic acid, *v*/*v*) (phase A) and acidified acetonitrile (1% acetic acid, *v*/*v*) (phase B) was prepared. The mixture was then filtered using a glass vacuum filtration device with 0.45 μm membrane filters from Millipore (Bedford, MA, USA) and degassed before use.

Chromatographic analysis conditions, as described in the literature [11,50], were either tested or adjusted accordingly. Following a sample injection volume of 10 μL, chromatographic separation was conducted using a Zorbax Extend C18 column (4.6 × 100 mm, 1.8 μm particle size, Agilent Technologies). The mobile phase gradient, applied at a flow rate of 1 mL/min, proceeded as follows: 10–25% phase B from 0 to 10 min; 25–60% phase B from 10 to 12 min; 60–80% phase B from 12 to 14 min; and 80–100% phase B from 14 to 18 min, maintained for 2 min, followed by a return to initial conditions in 2 min with a subsequent 3 min re-equilibration time. Throughout the whole analysis, the column oven temperature was kept constant at 40 °C, while DAD double on-line detection with optimal wavelengths of 240 nm and 280 nm was used.

The phenolic compounds in the analyzed extracts were identified by comparing their retention times and UV/vis data with those of authentic reference compounds and literature sources [11,50]. External standard curves were employed to determine the concentration of identified compounds. To do so, individual solutions of each pure standard were prepared at a concentration of 500 mg/L in methanol. Prior to injection into the HPLC system, all working standard solutions were filtered through a 0.22 μm nylon Clarinert^®^ syringe filter (Agela Technologies, Torrance, CA, USA).

For each standard, a calibration curve was generated with eleven different concentrations (0.5, 1, 2, 4, 8, 16, 32, 60, 125, 250 and 500 mL/L). If no pure standard was available, quantification was performed using the calibration curve of a similar compound.

Finally, the results for the dry samples were expressed in grams of phenolic compounds per kilogram of dry leaves (g/kg DW) and per equivalent dry weight (for fresh samples) (g/EDW), with all analyses performed in triplicate (n = 3).

### 3.6. Statistical Analysis

The results obtained in this study are reported as mean values ± standard deviation (SD) based on triplicate analyses conducted in two independent experiments (we examined two fresh samples and two samples for each drying method applied to each variety). For each sample, we conducted three extractions to analyze the compounds of interest. Each extract was subsequently analyzed three times, resulting in a total of eighteen analytical determinations per treatment and per variety, on average. Statistical analyses were performed using IBM SPSS Statistics software (SPSS for Windows, Version 20, SPSS Inc., Chicago, IL, USA). To assess the normality of data distribution and the homogeneity of variances, the Shapiro–Wilk and Levene tests were employed, respectively. ANOVA 2 was applied using an SNK test at a significance level of 5% to investigate the impact of olive leaf variety origin and the applied drying method.

## 4. Conclusions

This study sheds light on the effects of different dehydration methods (air-drying, oven-drying, solar-drying and freeze-drying) on the nutritional and bioactive compound content of olive leaves. The findings indicate varietal-specific responses to dehydration methods.

Regarding the fresh olive leaves, “Picholine Marocaine” displayed the highest content of total nitrogen and crude protein, while “Arbequina” had the highest level of total phenolic content. Fresh leaves of “Koroneiki” and “Menara” were found to be the richest in terms of total flavonoids. Oven-drying “Arbequina” leaves at 60 °C resulted in the highest levels of total nitrogen and crude protein, whereas oven-drying “Koroneiki” and “Picholine Marocaine” leaves at 70 °C yielded the highest nutrient levels. Solar-drying “Menara” leaves at 70 °C showed the highest nutritional content. In terms of total phenolic and flavonoid content, air-drying at room temperature proved to be the most suitable method for “Koroneiki” and “Picholine Marocaine” leaves, while oven-drying at 25 °C and air-drying were optimal for “Arbequina” leaves, respectively. Air-drying exhibited the highest total phenolic content, while freeze-drying “Menara” leaves demonstrated the highest total flavonoid content. A comprehensive analysis of the individual phenolic compounds revealed that oleuropein is the most abundant in all samples. In particular, freeze-drying of the leaves of “Arbequina” and “Picholine Marocaine” resulted in the highest oleuropein content, while air-drying and oven-drying of the leaves of “Koroneiki” and “Menara”, respectively, at 150 °C resulted in higher oleuropein contents. Although air-drying showed the most interesting results in the case of the “Koroneiki” samples in terms of phenolic content and the levels of the individual compounds, no single drying condition can be considered suitable for the other varieties. It is therefore obvious that the chosen drying method should be carefully selected, taking into account both the targeted compounds and the variety, in order to achieve optimal results. These findings highlight the significant impact of the drying process on the nutritional value and concentration of bioactive compounds in olive leaves. Moreover, they underscore the potential of olive leaves as valuable by-products for the development of a sustainable bioeconomy in the Moroccan olive industry.

To summarize, this study emphasizes the significance of recognizing the value of olive leaves and their potential to yield high-value-added compounds, especially considering their richness in various valuable compounds. The use of specific dehydration methods tailored to each variety and targeted compounds can help preserve or even enhance these compounds. The ongoing exploration and utilization of olive leaves can play a significant role in promoting sustainability and maximizing resource efficiency in the olive-growing sector.

## Figures and Tables

**Table 1:** Mean values and standard deviation for the total nitrogen and crude protein contents of fresh leaves from the four studied cultivars. All data expressed on a fresh weight basis. Different lower-case letters show significant differences between cultivars in fresh state (*p* < 0.05).

Main Factor	Cultivar	Total Nitrogen (%)	Crude Protein (%)	Number of Analyzed Extracts
Olive variety	“Picholine Marocaine”	0.98 ± 0.04 a	6.11 ± 0.23 a	6
	“Menara”	0.76 ± 0.02 b	4.78 ± 0.21 b	6
	“Arbequina”	0.88 ± 0.02 a	5.50 ± 0.12 a	6
	“Koroneiki”	0.92 ± 0.08 a	5.73 ± 0.50 a	6

**Table 2:** Mean values and standard deviation for the total phenolic and flavonoid content in fresh leaves from the four studied cultivars. Different lower-case letters show significant differences between cultivars in fresh state (*p* < 0.05). All data expressed on a fresh weight basis.

Main Factor	Cultivar	Total Phenolic Content (mg GAE/ g FW)	Total Flavonoid Content (mg QE/g FW)	Number of Analyzed Extracts
Olive variety	“Picholine Marocaine”	9.02 ± 2.05 a	2.75 ± 0.22 a	6
	“Menara”	14.85 ± 2.66 b	3.28 ± 0.08 a	6
	“Arbequina”	20.18 ± 0.95 c	3.26 ± 0.74 a	6
	“Koroneiki”	14.15 ± 2.91 b	3.28 ± 1.06 a	6

**Table 3:** Mean values and standard deviation for the total phenolic contents of fresh (expressed as mg GAE/g EDW) and dried (expressed as mg GAE/g DW) leaves from the four studied cultivars. Distinct capital letters represent significant differences between fresh state and drying methods for the same cultivar, while different lower-case letters show significant differences between cultivars in fresh state and for the same drying method (*p* < 0.05) (SNK test).

Drying Method	Cultivar			
	“Picholine Marocaine”	“Menara”	“Arbequina”	“Koroneiki”
**Fresh**	15.46 ± 0.24 aA	24.11 ± 0.22 bA	34.94 ± 0.20 cA	24.99 ± 0.23 bA
**Ambient-air-drying**	72.23 ± 0.21 aB	60.95 ± 0.20 bB	71.14 ± 0.11 cB	64.24 ± 0.16 dB
**Oven-drying at 25 °C**	48.16 ± 0.09 aC	42.98 ± 0.12 b	71.50 ± 0.20 cB	52.84 ± 0.18 dC
**Oven-drying at 50 °C**	45.00 ± 0.13 aD	33.07 ± 0.21 bC	35.23 ± 0.20 cA	40.30 ± 0.23 dD
**Oven-drying at 60 °C**	28.52 ± 0.12 aE	24.82 ± 0.19 bA	33.29 ± 0.32 cC	28.60 ± 0.25 aE
**Oven-drying at 70 °C**	31.61 ± 0.28 aF	27.55 ± 0.31 bE	26.59 ± 0.24 bD	21.55 ± 0.21 cF
**Oven-drying at 75 °C**	28.90 ± 0.24 aE	25.15 ± 0.22 bD	31.31 ± 0.30 cE	27.68 ± 0.12 dG
**Oven-drying at 80 °C**	19.79 ± 0.24 aG	41.38 ± 0.37 bF	30.27 ± 0.35 cF	22.59 ± 0.17 dH
**Oven-drying at 105 °C**	35.00 ± 0.20 aH	36.60 ± 0.38 bG	37.71 ± 0.24 bG	31.67 ± 0.41 cI
**Oven-drying at 120 °C**	34.39 ± 0.23 aH	39.05 ± 0.14 bH	47.78 ± 0.17 cH	37.15 ± 0.26 dJ
**Oven-drying at 150 °C**	43.03 ± 0.20 aI	39.56 ± 0.19 bH	41.53 ± 0.12 cI	49.26 ± 0.23 dK
**Freeze-drying**	39.42 ± 0.20 aJ	45.32 ± 0.16 bI	42.57 ± 0.16 cJ	40.86 ± 0.20 dD
**Solar-drying at 50 °C**	18.48 ± 0.63 aK	21.78 ± 0.80 bJ	20.92 ± 0.41 bK	17.75 ± 0.41 aL
**Solar-drying at 60 °C**	21.37 ± 0.22 aL	25.25 ± 0.39 bD	24.83 ± 0.61 bL	28.87 ± 0.61 cE
**Solar-drying at 70 °C**	35.20 ± 0.16 aM	41.91 ± 0.65 bF	24.83 ± 0.61 cL	31.04 ± 0.82 dI
**Solar-drying at 80 °C**	27.66 ± 0.53 aN	33.12 ± 0.49 bC	30.32 ± 0.61 cF	27.14 ± 0.61 aG

**Table 4:** Mean values and standard deviations for the total flavonoid content of fresh (expressed as mg QE/g EDW)) and dried (expressed as mg QE/g DW) leaves from the four studied cultivars. Distinct capital letters represent significant differences between fresh state and drying methods for the same cultivar, while different lower-case letters show significant differences between cultivars in fresh state and for the same drying method (*p* < 0.05) (SNK test).

Drying Method	Cultivar			
	“Picholine Marocaine”	“Menara”	“Arbequina”	“Koroneiki”
**Fresh**	4.67 ± 0.37 aA	5.29 ± 0.13 aA	5.61 ± 1.28 aA	5.75 ± 1.86 aA
**Ambient-air-drying**	20.54 ± 0.30 aB	21.92 ± 0.22 abB	24.01 ± 1.36 bcB	25.45 ± 0.06 cB
**Oven-drying at 25 °C**	18.89 ± 0.54 aC	18.43 ± 0.32 aC	22.64 ± 0.43 bBC	21.41 ± 0.22 bC
**Oven-drying at 50 °C**	15.75 ± 0.22 aD	14.22 ± 0.22 aD	15.37 ± 3.14 aD	20.57 ± 2.27 aC
**Oven-drying at 60 °C**	6.17 ± 0.32 abE	5.17 ± 0.04 aA	6.13 ± 0.54 abA	7.16 ± 0.65 bADE
**Oven-drying at 70 °C**	6.30 ± 0.26 abE	8.06 ± 0.19 bE	5.78 ± 0.43 aA	5.77 ± 0.93 aA
**Oven-drying at 75 °C**	5.84 ± 0.17 aE	8.31 ± 0.11 aE	7.16 ± 0.65 aAE	8.03 ± 1.10 aADE
**Oven-drying at 80 °C**	6.06 ± 0.13 aE	17.82 ± 1.41 bCF	6.43 ± 0.35 aA	6.55 ± 0.61 aAE
**Oven-drying at 105 °C**	15.67 ± 0.76 aD	17.28 ± 0.87 aCF	18.35 ± 3.03 aCD	16.97 ± 1.73 aFG
**Oven-drying at 120 °C**	13.76 ± 0.43 aF	16.59 ± 0.76 aF	17.51 ± 1.19 aD	16.13 ± 1.41 aG
**Oven-drying at 150 °C**	19.58 ± 0.87 aC	18.28 ± 0.11 aC	20.34 ± 2.6 aB	19.12 ± 0.43 aCF
**Freeze-drying**	17.28 ± 0.65 aG	22.64 ± 0.65 bB	22.26 ± 2.27 abB	21.18 ± 0.76 abC
**Solar-drying at 50 °C**	5.48 ± 0.09 aAE	6.64 ± 0.17 bG	5.12 ± 0.24 aA	5.17 ± 0.13 aA
**Solar-drying at 60 °C**	6.09 ± 0.17 aE	7.51 ± 0.07 bEG	5.43 ± 0.06 cA	9.64 ± 0.09 dDE
**Solar-drying at 70 °C**	3.38 ± 0.06 aH	4.10 ± 0.09 bA	10.89 ± 0.09 cE	8.46 ± 0.06d ADE
**Solar-drying at 80 °C**	9.90 ± 0.07 aI	12.08 ± 0.08 bH	8.77 ± 0.15 cAE	10.34 ± 0.09 dD

## Data Availability

The authors confirm that all data underlying the findings of this work are available within this manuscript and its Supplementary File. Raw data that support the outcomes of this study are available from the corresponding authors, upon reasonable request.

## Supplementary Materials

The following supporting information can be downloaded at: https://www.mdpi.com/article/10.3390/plants13141961/s1, Table S1: Results of the ANOVA 2 test on the obtained data of total nitrogen, crude protein, total polyphenol and total flavonoid content of the samples from the investigated varieties and applying the selected drying methods. Figure S1. Representative chromatograms obtained by HPLC analysis of the “Picholine Marocaine” fresh and dried samples under the different investigated methods. Figure S2. Contents of phenolic compounds detected and quantified in fresh samples of the four studied cultivars. Figure S3. Evolution of the weight of the samples as a function of time by applying ambient-air-drying and oven-drying at different temperatures.
