# Supplementary material for: Nutrient and Bioactive Fraction Content of Olea europaea L. Leaves: Assessing the Impact of Drying Methods in a Comprehensive Study of Prominent Cultivars in Morocco
Source: Plants (Basel). 2024 Jul 17;13(14):1961. doi: 10.3390/plants13141961 (PMC11281108; doi:10.3390/plants13141961)
Supplement: Supplementary file 1 [file plants-13-01961-s001.zip › plants-3101841-supplementary.pdf]

Table S1. Results of the ANOVA 2 test on the obtained data of total nitrogen, crude protein, total polyphenols and total flavonoid content of the samples from the investigated varieties and applying the selected drying methods.

Parameter	Factor	Significance
Total nitrogen content	Variety	0.00
	Drying method	0.00
	Variety * drying method	0.00
Crude protein content	Variety	0.00
	Drying method	0.00
	Variety * drying method	0.00
Total phenolic content	Variety	0.00
	Drying method	0.00
	Variety * drying method	0.00
Total flavonoid content	Variety	0.00
	Drying method	0.00
	Variety * drying method	0.00

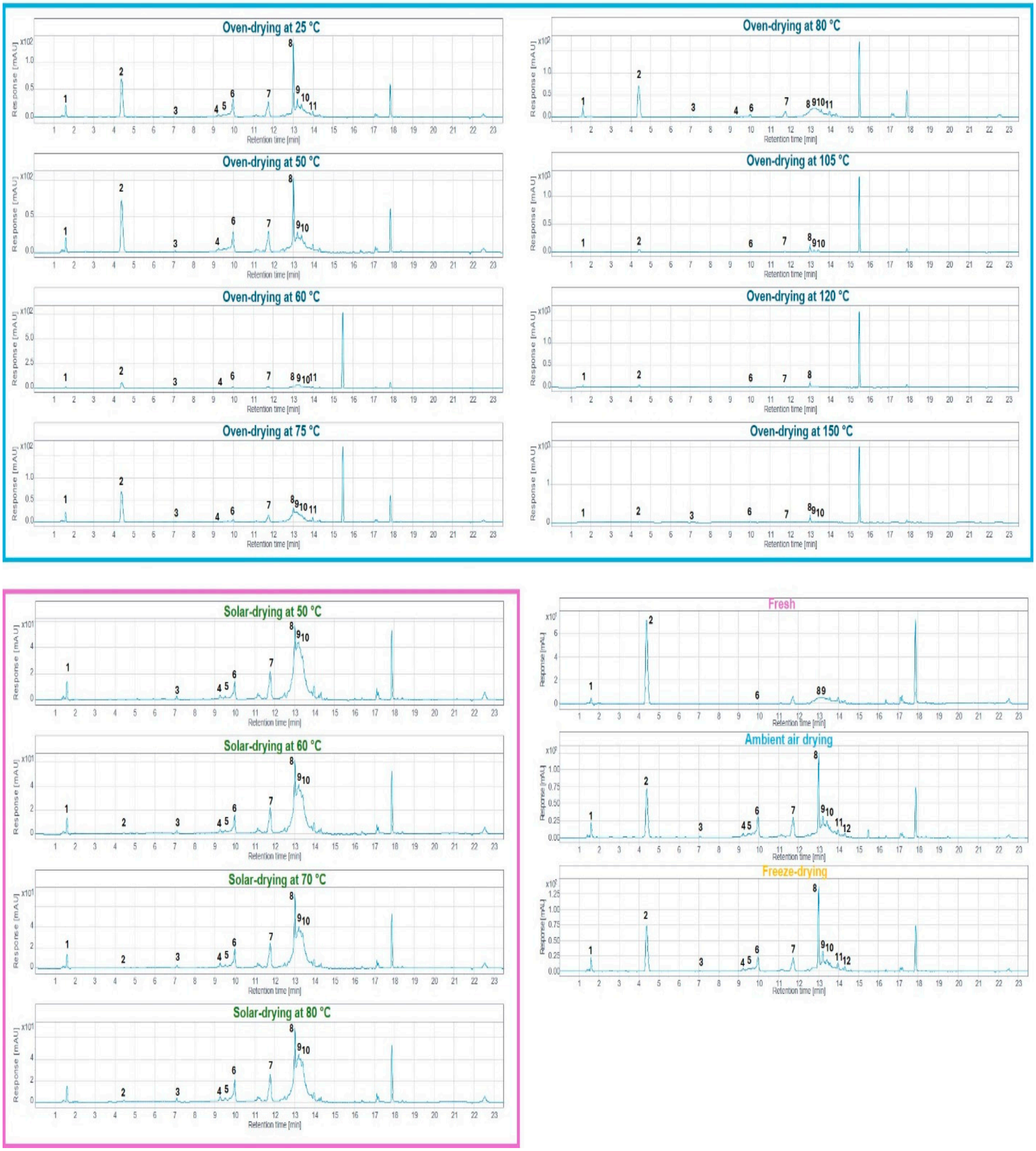

Figure S1. Representative chromatograms obtained by HPLC analysis of the "Picholine Marocaine" fresh and dried samples under the different investigated methods. Abbreviation: 1: quinic acid; 2: hydroxytyrosol glucoside; 3: rutin; 4: luteolin 7-*O*-glucoside; 5: verbascoside; 6: hydroxytyrosol acetate; 7: apigenin-7-*O*-glucoside; 8: oleuropein; 9: luteolin; 10: pinoresinol; 11: apigenin.

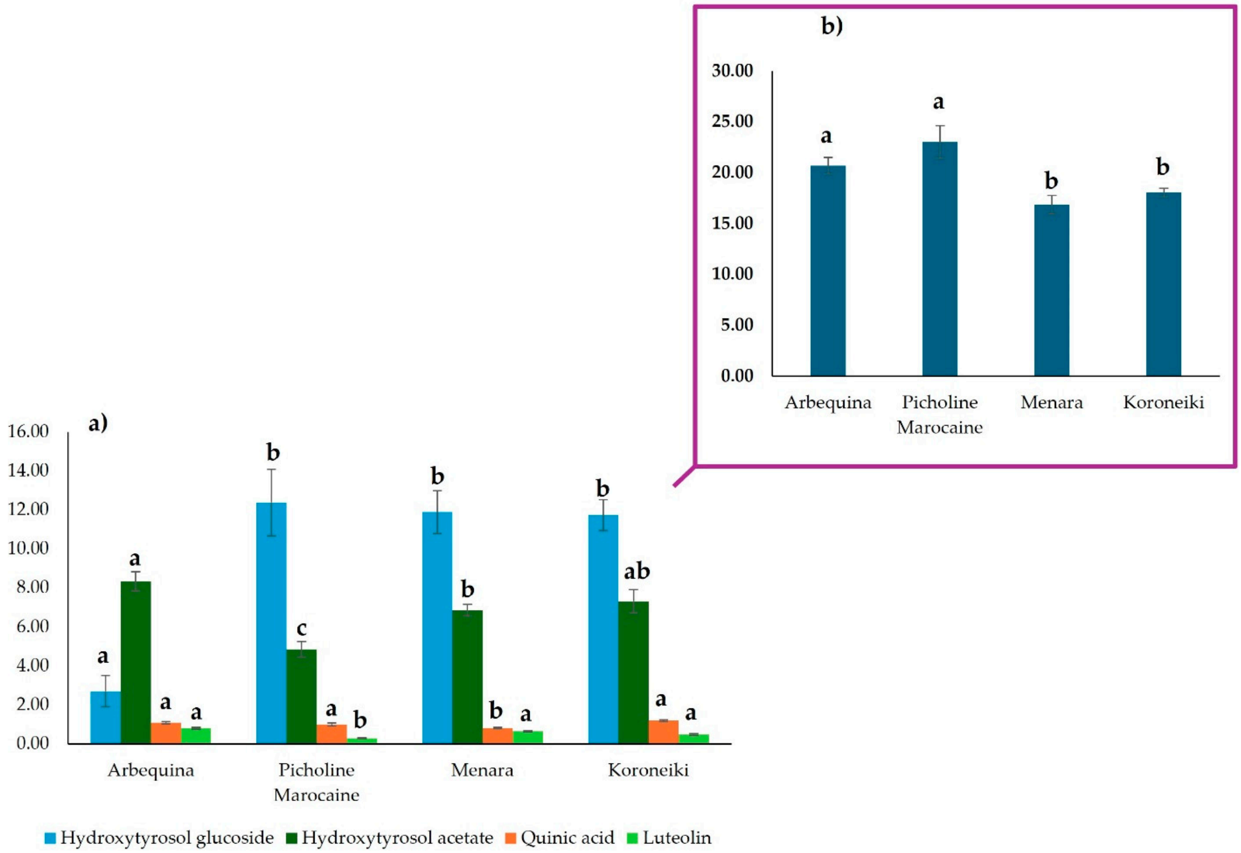

Figure S2. Contents of phenolic compounds detected and quantified in fresh samples of the four studied cultivars: a) contents of quinic acid, hydroxytyrosol glucoside, hydroxytyrosol acetate and luteolin expressed as mg/kg FW; b) contents of oleuropein expressed as mg/kg FW. Different lower-case letters show significant differences between cultivars at fresh state ($p < 0.05$).

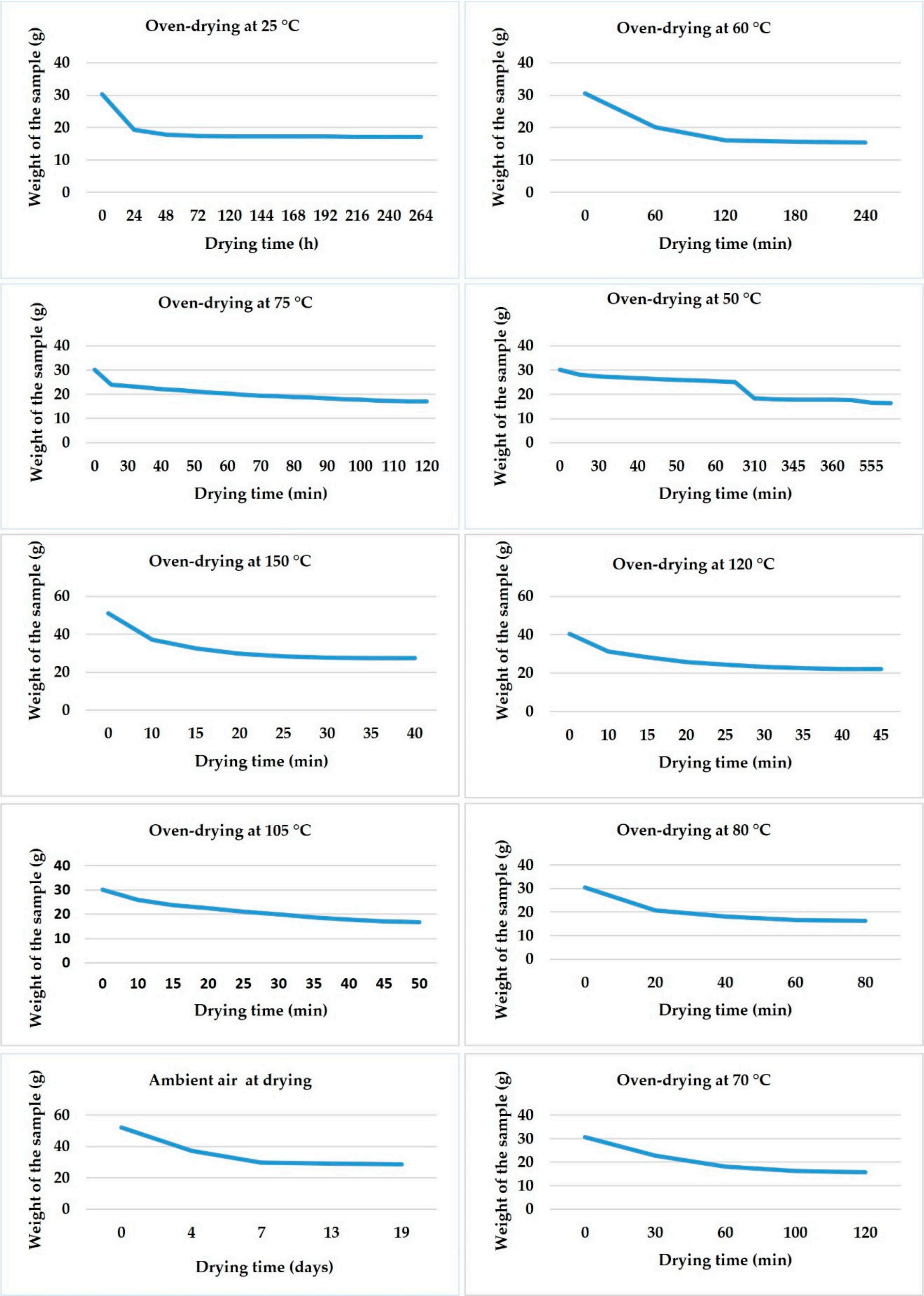

Figure S3. Evolution of the weight of the samples as a function of time by applying ambient air drying and oven-drying at different temperatures.
